# Parasite infection of public databases: a data mining approach to identify apicomplexan contaminations in animal genome and transcriptome assemblies

**DOI:** 10.1186/s12864-017-3504-1

**Published:** 2017-01-19

**Authors:** Janus Borner, Thorsten Burmester

**Affiliations:** Institute of Zoology, Biocenter Grindel, University of Hamburg, Martin-Luther-King-Platz 3, D-20146 Hamburg, Germany

**Keywords:** Apicomplexa, Contamination, Database analysis, Phylogeny, Coccidia, Piroplasmida, Gregarinasina, Haemosporida, Malaria, Parasites

## Abstract

**Background:**

Contaminations from various exogenous sources are a common problem in next-generation sequencing. Another possible source of contaminating DNA are endogenous parasites. On the one hand, undiscovered contaminations of animal sequence assemblies may lead to erroneous interpretation of data; on the other hand, when identified, parasite-derived sequences may provide a valuable source of information.

**Results:**

Here we show that sequences deriving from apicomplexan parasites can be found in many animal genome and transcriptome projects, which in most cases derived from an infection of the sequenced host specimen. The apicomplexan sequences were extracted from the sequence assemblies using a newly developed bioinformatic pipeline (ContamFinder) and tentatively assigned to distinct taxa employing phylogenetic methods. We analysed 920 assemblies and found 20,907 contigs of apicomplexan origin in 51 of the datasets. The contaminating species were identified as members of the apicomplexan taxa Gregarinasina, Coccidia, Piroplasmida, and Haemosporida. For example, in the platypus genome assembly, we found a high number of contigs derived from a piroplasmid parasite (presumably *Theileria ornithorhynchi*). For most of the infecting parasite species, no molecular data had been available previously, and some of the datasets contain sequences representing large amounts of the parasite’s gene repertoire.

**Conclusion:**

Our study suggests that parasite-derived contaminations represent a valuable source of information that can help to discover and identify new parasites, and provide information on previously unknown host-parasite interactions. We, therefore, argue that uncurated assembly data should routinely be made available in addition to the final assemblies.

**Electronic supplementary material:**

The online version of this article (doi:10.1186/s12864-017-3504-1) contains supplementary material, which is available to authorized users.

## Background

Contaminations by DNA from non-target organisms are a common problem in next-generation sequencing projects [1–3]. If these contaminants are not flagged and remain in the datasets after sequence assembly and deposition into public databases, subsequent analyses of the datasets may yield confusing results and may lead to false conclusions [4, 5]. Various computational methods have been developed that are highly efficient at identifying and removing common contaminants, such as DNA from cloning vectors or human DNA, before sequence assembly [6, 7]. By contrast, contaminations by DNA from other sources, e.g. via aerosol contamination in the laboratory or at the sequencing center, are notoriously difficult to identify.

Another potential source of contamination are pathogens present in the source material [8–10]. In genome projects of wild animals, it is virtually impossible to rule out infection by an unknown pathogen before sequencing. The development of bioinformatic approaches to identify contamination by pathogens is therefore of great importance. Most existing tools aim to assign individual reads to taxonomic groups without prior assembly. As the amount of read data in next-generation sequencing (NGS) projects is enormous and the reads are short and of low quality, the programs either rely on near exact matches at the nucleotide level [11], or employ smaller databases containing only selected marker genes [12] or genes that are specific to certain clades [13]. The former approach is not suited for the identification of contaminations by parasites for which only distantly related species are available in the public databases, whereas the latter approach is especially useful for quantitative estimates of genome abundance but can only find a small number of predefined genes. The program PathSeq [14], which was developed to identify microorganisms by deep sequencing of human tissue, uses a different approach by first subtracting all reads derived from the human host. However, this is obviously only feasible when high-quality genome data is already available for the host species.

While previous approaches have mostly focused on the removal of contaminating sequences, the identification of parasite-derived contaminations may also enable the discovery of novel parasite taxa and shed light on previously unknown host-parasite associations. For example, a recent study by Orosz [10] has highlighted that contaminations by parasite DNA may also represent a source of information. By searching published whole genome shotgun assemblies from various animal taxa for a protein (apicortin) that is characteristic for apicomplexan parasites but absent in animals (Eumetazoa), the author identified sequences from apicomplexan parasites in two animal genome assemblies from the northern bobwhite (*Colinus virginianus*) and the bat *Myotis davidii*. Data mining of genome assemblies from infected hosts may produce large amounts of genomic data from pathogens that are not yet represented in the public databases.

Members of the protozoan phylum Apicomplexa are obligate parasites that may cause serious illnesses in humans and animals. For example, five distinct species of the genus *Plasmodium* are the causative agents of human malaria and, as such, pose one of the greatest threats to public health [15]. While the gregarines (Gregarinasina) only infect invertebrates, members of the apicomplexan taxa Coccidia and Piroplasmida are responsible for numerous infectious diseases in wild and domesticated animals, such as coccidiosis and babesiosis, resulting in considerable animal health problems and economic losses [16].

Here we present a bioinformatic pipeline (ContamFinder) to identify parasite contamination in NGS assembly data and extract genetic sequences derived from the contaminating parasite. Phylogenetic methods were employed to assign the sequences to apicomplexan taxa. In total, we found contaminating sequences of apicomplexan origin in 51 genome and transcriptome assemblies. The amount of parasite-derived coding sequences varies greatly among the contaminated assemblies from just a few contigs to a significant amount of the parasite’s gene repertoire.

## Methods

### Data selection

We downloaded all available metazoan genome and transcriptome assemblies from the Whole Genome Shotgun (WGS) [17] and Transcriptome Shotgun Assembly (TSA) [18] databases. As no gene predictions were available for the genome sequences from *Ascogregarina taiwanensis* (WGS prefix ABJQ01), the contigs were processed alongside the metazoan assemblies using the pipeline described below in order to obtain predicted protein sequences for this taxon.

### Extraction of parasite-derived sequences

**Fig. 1:**
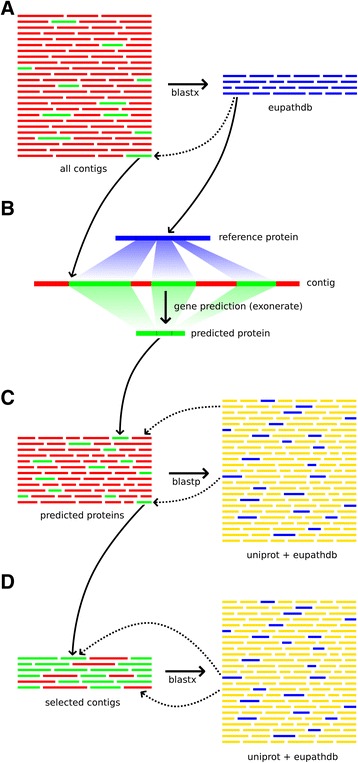
Schematic overview of the ContamFinder pipeline. **a** All contigs from an assembly were searched against apicomplexan proteomes from the Eukaryotic Pathogen Database (EuPathDB [19, 20]). Sequences without significant hit were discarded. **b** Amino acid sequences were predicted using the best hitting apicomplexan protein. Low complexity regions and repeats in the sequence were masked. **c** The predicted amino acid sequences were searched against the EuPathDB and UniProt database. Sequences with the best hit outside of Apicomplexa were discarded. **d** Unprocessed contigs corresponding to the hits from the previous step were searched against the EuPathDB and UniProt databases. Sequences that had their best hit outside of Apicomplexa were discarded. Contigs and sequence regions that were kept and used in the next step are shown in green; sequences that were discarded are denoted in red. Parasite-derived proteins in the search database are shown in blue, others in yellow

In the first step (Fig. 1a) of the ContamFinder pipeline, all contigs from each assembly were subjected to a search against all apicomplexan proteomes from the Eukaryotic Pathogen Database (EuPathDB) [19, 20]. All searches were performed employing GHOSTX [21] based on its high performance (Table 1) in a test run on the transcriptome assembly of the domestic goat, *Capra hircus* (TSA prefix GAOJ01), and the genome assembly of the white-tailed deer, *Odocoileus virginianus* (WGS prefix AEGY01), but ContamFinder also supports output from BLAST+ [22] and RAPSearch2 [23]. Sequences that showed significant sequence similarity (E-value cut-off: 1e-10; see below) to a parasite protein were analyzed further; the rest was discarded. By searching against a relatively small database (compared to UniProt) first, and by the subsequent removal of all contigs without sequence similarity, we massively reduced the amount of sequences that needed to be searched against the UniProt database. However, as highly conserved genes from a metazoan organism may have significant sequence similarity to parasite genes, this initial selection contained large amounts of false positives. Preliminary analyses showed that blastx-style searches of the remaining contigs against the UniProt database would still be too slow for large numbers of genome assemblies, which may contain very long contigs.

**Table 1:** Performance of the ContamFinder pipeline employing three different sequence similarity search tools compared to an all-vs-all blastx search

	Assembly type	Assembly size	all-vs-all blastx search (BLAST+)	ContamFinder (BLAST+)	ContamFinder (RAPsearch2)	ContamFinder (GHOSTX)
*Capra hircus* (GAOJ01)	transcriptome	25.1 Mb	82 h 14 min 439 hits	15 h 57 min 418 hits	40 min 396 hits	25 min 405 hits
*Odocoileus virginianus* (AEGY01)	genome	14.3 Mb	36 h 9 min 127 hits	1 h 12 min 122 hits	8 min 104 hits	3 min 98 hits

To further improve the performance, the amino acid sequence encoded in each of the potentially parasite-derived contigs was predicted in the second step (Fig. 1b). Gene prediction was performed by the program Exonerate [24] using the best hitting protein from EuPathDB as guide (with “full refinement” of the alignments, employing the protein2dna model for transcriptome data and the protein2genome model for genome data). Subsequently, regions of low complexity or repeats in the amino acid sequence were masked by the SEG filter from the BLAST+ package.

In the third step (Fig. 1c), the predicted amino acid sequences were searched against all complete proteomes from the UniProt database. Sequences that had their best hit against a protein from an apicomplexan species were extracted for further analysis; the rest was discarded. In preliminary analyses, we found several false positive hits caused by falsely annotated proteins in the UniProt database that were in fact derived from the parasite’s host. Therefore, we removed all protein sequences annotated as apicomplexan and replaced them with the genome-based proteome predictions available in the well-curated EuPathDB. Vice versa, undetected parasite contamination in a genome or transcriptome assembly may have led to parasite proteins being falsely assigned to the host species in the Uniprot database. This would cause similarity searches to produce false-negative results when analyzing the affected assembly. To avoid discarding such contaminants, hits against sequences from the source species were ignored.

Because the predicted amino acid sequences were obtained by using the best hitting parasite protein as a guide sequence, they may be biased towards showing a high similarity to this protein. Therefore, in the final step of the pipeline (Fig. 1d), we searched the unprocessed nucleotide contigs corresponding to the hits from the previous step against the same database (UniProt + EuPathDB). Again, sequences that had their best hits against proteins of non-apicomplexan origin were discarded.

For a few sequencing projects, the WGS and TSA databases contained multiple assemblies that were based on the same raw sequencing data. In these cases, we only kept the results from the assembly with the highest number of hits. All analyses were run on the high-performance computing cluster of the Regionales Rechenzentrum (RRZ), University of Hamburg, employing dual CPU compute nodes, each equipped with two Intel Xeon E5-2630v3 CPUs.

### Orthology prediction and multiple sequence alignment

Predicted proteome data derived from all available apicomplexan and chromerid genomes (maximum one per species) were obtained from EuPathDB and assigned to ortholog groups based on their OrthoMCL [25] annotation available in EuPathDB. Ortholog groups were required to contain sequences from at least three of the six major taxonomic groups (Chromerida, Gregarinasina, *Cryptosporidium*, Coccidia, Piroplasmida, Haemosporida). To obtain a dataset of unambiguous one-to-one orthologs, groups that contained more than one sequence from the same proteome were discarded. All predicted parasite proteins from the metazoan sequence assemblies were assigned to these orthologous groups by OrthoMCL. Genes with a taxon coverage of less than 30% were removed to reduce the amount of missing data in the final dataset, resulting in 1,420 genes from 67 taxa (dataset 1). As this dataset was too large for Bayesian tree inference, a reduced dataset was generated (minimum taxon coverage of 70% for each gene, minimum of 10 genes per taxon). This dataset comprises 301 genes from 49 taxa (dataset 2). Each group of orthologous proteins was aligned individually using MAFFT L-INS-i v7.013 [26]. Poorly aligned sections of the amino acid alignments were eliminated by Gblocks v0.91b [27] (settings: −b1 = [50% of the number of sequences + 1] -b2 = [85% of the number of sequences] -b3 = 8 -b4 = 10 –b5 = h). The final concatenated super alignment comprised 216,613 amino acid (aa) positions (57.0% missing data/gaps) for dataset 1 and 66,467 aa (31.3% missing data/gaps) for dataset 2.

### Phylogenetic analyses

A maximum likelihood (ML) tree was calculated by RAxML 8.2.8 [28] based on dataset 1 using the LG amino acid substitution matrix [29] with empirical amino acid frequencies and assuming a gamma distribution of rates across sites. Bayesian tree inference was performed by PhyloBayes MPI 1.7b [30] based on dataset 2. Eight independent chains were run under the CAT model of sequence evolution [31] with four discrete gamma categories. Every 10^th^ cycle was sampled, and the chains were stopped after 10,000 cycles. After 2500 cycles, all model parameters had entered the stationary phase. A majority rule consensus tree was calculated discarding the first 25% of samples as burn-in from all eight runs. The comparison of bipartitions showed minimal discrepancy among chains (maxdiff value = 0.11) indicating that all eight runs had converged in tree space. Additionally, the bootstrap support values from a ML analysis of dataset 2 (using the same parameters as described above) were mapped onto the Bayesian consensus tree. The resulting trees based on analyses of both datasets were rooted with the chromerid taxa *Chromera velia* and *Vitrella brassicaformis*.

## Results and discussion

### A data mining approach to identify parasite contamination

The goal of this study was (*i.*) to quantify the extent of contamination by apicomplexan parasites in animal genome and transcriptome assemblies and (*ii.*) to extract as much useful sequence information of parasite origin from these assemblies. A naive, brute force approach to the identification of contaminating sequences might employ a simple blastx query, i.e. searching all contigs of a genome project against a database containing the entire record of publicly available proteomes across all taxa. In a second step, contigs that show the highest similarity to sequences from parasite species could then be extracted as putative contaminants. While such an approach might be feasible for a small number of contigs, it is highly inefficient. The computational resources required to apply this procedure to all available animal genomes exceed even the limits of high-performance computer centers because blastx-style (translated nucleotide vs. protein) searches against large protein databases such as Uniprot are very computationally intensive, especially when using large genomic contigs as query.

In our approach, we drastically reduced the computational complexity of this problem by first filtering the genome data to extract only those contigs that show significant sequence similarity to proteins from apicomplexan parasites (Fig. 1a). By incorporating homology-based gene prediction into the process of contamination identification in the next step (Fig. 1b), we were able to further improve the performance of the search strategy. This allowed us to perform protein vs. protein searches against the UniProt database first (Fig. 1c), which is significantly faster than using the full-length nucleotide contigs as query. Additionally, this step provides high-quality amino acid data for all identified contaminating sequences, which can subsequently be used, e.g., for phylogenetic analyses. After removal of all contigs with a best hit outside of Apicomplexa, the final nucleotide vs. protein searches were performed on a minimal subset of suspect contigs to assess whether they were indeed of apicomplexan origin (Fig. 1d).

### Comparison of sequence similarity search tools

**Fig. 2:**
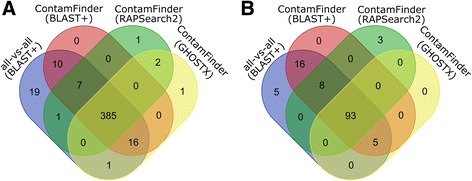
Venn diagrams showing shared and unique hits from analyses using different search strategies on the assemblies of *Capra hircus* (**a**) and *Odocoileus virginianus* (**b**)

To assess whether the performance gains achieved by the ContamFinder pipeline would be sufficient for large-scale analysis of all available genome and transcriptome assembly data, we compared the performance of ContamFinder (employing BLAST+ as search engine) to a naive all all-vs-all blastx search against the UniProt database. Analyses were performed on the transcriptome assembly of the domestic goat, *Capra hircus* (TSA prefix GAOJ01), which contains sequences of coccidian origin, and the comparatively small (14.3 Mb) genome assembly of the white-tailed deer, *Odocoileus virginianus* (WGS prefix AEGY01), infected with a piroplasmid parasite. In both analyses, ContamFinder was able to recover >95% of the hits identified in the all-vs-all blastx search (Table 1) while increasing the speed of the analysis 5-fold for the transcriptome assembly and 30-fold for the genome assembly. The difference in performance gain can be explained by the large amount of non-coding sequence regions in genome data which slow down the blastx search and which are discarded by ContamFinder during the gene prediction step (Fig. 1b). Considering that the total amount of sequence data available from genome assemblies far exceeds that from transcriptome assemblies, these performance metrics are highly favorable for the large scale application of ContamFinder on all available assembly data. However, as most genome assemblies contain much larger amounts of sequence data (in the order of Gb) than the small dataset that was used as a benchmark, we decided to investigate whether the use of alternative amino acid similarity search algorithms could further improve the speed of the analyses. We compared the performance of three local alignment tools (BLAST+ [22], RAPSearch2 [23], GHOSTX [21]). While BLAST+ identified slightly more parasite-derived contigs in both assemblies, GHOSTX and RAPSearch2 were able to speed up the search significantly with an acceptable impact on sensitivity (Table 1). As the amount of computational time required for BLAST+-based analyses of large genome assemblies becomes prohibitively large, we decided to perform all further analyses using GHOSTX, which reduced the run time of ContamFinder 24-fold compared to the BLAST+-based ContamFinder analysis and more than 700-fold compared to a simple blastx all-vs-all search (Table 1). Because in the last step of the pipeline ContamFinder basically performs a blastx all-vs-all search with a drastically reduced query pool (Fig. 1d), all hits from the BLAST+-based ContamFinder analysis were also found in the simple blastx all-vs-all search. When using GHOSTX or RAPSearch2 as the search tool, small numbers (three in each case) of additional hits were found (Fig. 2). Closer inspection of these hits showed that all of them constitute valid parasite-derived contaminations.

### Assemblies from aquatic metazoans contain high amounts of protozoan contaminants

For the analysis of apicomplexan parasite contaminations in public databases, we downloaded all available metazoan genome and transcriptome assemblies from the Whole Genome Shotgun (WGS; 658 assemblies) and Transcriptome Shotgun Assembly (TSA; 703 assemblies) databases. Preliminary analyses showed multiple putative apicomplexan species present in most genomes from aquatic species (with aquatic mammals being a notable exception). This may be caused either by infections with multiple parasite species or by contamination of the samples with free-living alveolates closely related to Apicomplexa (e.g. Chromerida). Because the goal of this study was to evaluate and reliably classify the contaminating parasites using multi-gene phylogenetic analyses, which require that each sample only contain a single species, we decided to discard all assemblies from non-mammalian aquatic species and to focus on terrestrial animals. Further analysis of parasite contamination in genomes and transcriptomes from aquatic animals might yield valuable insight into host-parasite associations in aquatic ecosystems.

### Genome and transcriptome assemblies of terrestrial animals may contain large amounts of parasite-derived contigs

After removal of 349 assemblies from aquatic species and 59 assemblies from metazoan endoparasites, we performed analyses on the remaining 953 assemblies from terrestrial animals and aquatic mammals (583 Gb). We found contigs of putatively apicomplexan origin in 85 genome and transcriptome projects. The number of identified parasite-derived contigs varied greatly among the contaminated assemblies (Table 2). While most assemblies contained only low to moderate numbers of parasite-derived sequences, we found massive amounts of apicomplexan sequences in the genome assemblies of the northern bobwhite, *Colinus virginianus* (WGS prefix AWGU01; 4,081 contigs), and the duck-billed platypus, *Ornithorhynchus anatinus* (WGS prefix AAPN01; 1,397 contigs). We also found a large number of parasite-derived contigs in the transcriptome assemblies of the oriental tobacco budworm, *Helicoverpa assulta* (TSA prefix GBTA01; 8,347 contigs), the cotton bollworm, *Helicoverpa armigera* (TSA prefix GBDM01; 1,137 contigs) and the stalk-eyed fly, *Teleopsis dalmanni* (TSA prefix GBBP01; 919 contigs). These numbers show that our approach is valid for both genome and transcriptome data. As we were mostly interested in conserved genes for use in phylogenetic analyses, we performed all sequence similarity searches with a strict E-value cut-off of 1e-10. Lowering the E-value cut-off would certainly increase the amount of identified parasite sequences – though at the cost of an increased risk of false positives.

**Table 2:** Numbers of parasite-derived contigs in publicly available genome and transcriptome assemblies

Host species	WGS/TSA ID	Assembly type	# parasite-derived contigs	# sequences in dataset 1	# sequences in dataset 2
*Helicoverpa assulta*	GBTA01	transcriptome	8347	370	208
*Colinus virginianus*	AWGU01	genome	4013	793	244
*Colinus virginianus* ^a^	AWGT01	genome	3098	-	-
*Ornithorhynchus anatinus* ^c^	AAPN01	genome	1397 (119)	540	178
*Helicoverpa armigera*	GBDM01	transcriptome	1137	160	102
*Teleopsis dalmanni*	GBBP01	transcriptome	919	339	171
*Capra hircus*	GAOJ01	transcriptome	405	107	63
*Annulipalpia sp*.	GATX01	transcriptome	226	81	57
*Gorilla gorilla gorilla* ^c^	CABD02 (CABD03)	genome	148 (3)	33	15
*Camelus dromedarius*	GADZ01	transcriptome	148	35	25
*Anolis carolinensis*	GBBS01	transcriptome	120	54	33
*Anolis carolinensis* ^a^	GAFN01	transcriptome	119	-	-
*Dendroctonus frontalis* ^b^	GAFI01	transcriptome	114	-	-
*Dastarcus helophoroides*	GBCX01	transcriptome	104	29	21
*Odocoileus virginianus*	AEGY01	genome	98	34	11
*Odocoileus virginianus* ^a^	AEGZ01	genome	98	-	-
*Motis davidii*	ALWT01	genome	66	9	-
*Anolis carolinensis* ^a^	GAFD01	transcriptome	62	-	-
*Orchesella cincta*	GAMM01	transcriptome	61	30	27
*Ixodes ricinus* ^b^	GADI01	transcriptome	56	-	-
*Corydalinae sp*.	GADH01	transcriptome	41	18	-
*Pseudomasaris vespoides*	GAXQ01	transcriptome	39	18	17
*Camelus dromedarius* ^a^	GADZ01	transcriptome	24	-	-
*Ixodes scapularis*	ABJB01	genome	26	7	-
*Homo sapiens*	AADC01	genome	24	6	-
*Polyxenus lagurus*	GBKF01	transcriptome	21	12	-
*Dendroctonus ponderosae*	GAFW01	transcriptome	15	6	-
*Amblyomma americanum*	GAGD01	transcriptome	10	4	-
*Carduelis chloris*	GBCG01	transcriptome	8	-	-
*Capra hircus*	GAOE01	transcriptome	8	-	-
*Ixodes ricinus*	GANP01	transcriptome	7	5	-
*Camelus bactrianus*	GAEY01	transcriptome	7	2	
*Dendroctonus ponderosae* ^a^	GAFX01	transcriptome	6	-	-
*Chrysochloris asiatica*	AMDV01	genome	5	2	-
*Cuculus canorus*	JNOX01	genome	5	2	-
*Bos mutus*	AGSK01	transcriptome	5	1	-
*Nevrorthus apatelios*	GACU01	transcriptome	4	3	-
*Fulmarus glacialis*	JJRN01	genome	4	2	-
*Forficula auricula*	GAAX01	transcriptome	4	3	-
*Serinus canaria*	CAVT01	genome	3	2	-
*Capra hircus*	GAFC01	transcriptome	3	2	-
*Balaenoptera bonaerensis*	BAUQ01	genome	2	1	-
*Blattela germanica*	GBID01	transcriptome	2	-	-
*Folsomia candida*	GAMN01	transcriptome	2	-	-
*Carabus granulatus*	GACW01	transcriptome	1	-	-
*Capra hircus*	GAOG01	transcriptome	1	-	-
*Nemurella pictetii*	GAAV01	transcriptome	1	-	-
*Anolis carolinensis*	GADN01	transcriptome	1	-	-
*Phaedon cochleariae*	GAPU01	transcriptome	1	-	-
*Gluvia dorsalis*	GDAP01	transcriptome	1	-	-
*Rhipicephalus microplus*	ADMZ02	genome	1	-	-

^a^Assembly was not used in phylogenetic analyses because it is based on the same raw data as another assembly

^b^Assembly was not used in phylogenetic analyses because it contains sequences from multiple parasite species

^c^Data based on a superseded assembly version; the number of parasite-derived contigs in the current version is given in parentheses

### False-positive hits may be caused by low sequence complexity or high conservation

In 35 assemblies, only a single hit was found. Closer inspection revealed that 28 of the single hits were false positives, which were either due to highly conserved proteins (20 hits), such as ubiquitin or tubulin, or caused by repetitive sequence patterns (8 hits) that had not been removed by the low complexity filtering step. The exclusion of these conserved proteins from the reference proteomes and the application of advanced filtering methods [32, 33] might alleviate this problem in the future. Among the 50 assemblies with more than one hit, another five were found to be based on small numbers of false positives (2–5 hits). However, the total number of hits identified as false-positive (43 contigs) pales in comparison to the total number of hits from assemblies that are indeed contaminated by parasite sequences (20,907 contigs). Of course, we cannot rule out that the extracted data from these assemblies also contain small numbers of erroneously identified contigs. Large fractions of the extracted contigs (between 20% and 80%, depending on fragmentation of the assembly) also had significant hits against proteins from non-Apicomplexan species. This is to be expected as the majority of apicomplexan genes have detectable homologs in other eukaryotes, especially in the closely related chromerids [34]. We inspected at least 20 (or as many as available) of these contigs for each assembly using single-gene phylogenetic analyses and sequence similarity searches and found no evidence of false-positive hits.

### Unambiguous parasite contaminations were found in 51 assemblies

In total, 51 assemblies contained unambiguous contamination by apicomplexan parasites. However, six assemblies were based, at least in part, on the same raw sequencing data or source specimen as other assemblies in our dataset and were therefore removed. Of the remaining 45 assemblies, 11 did not contain sequences that could be assigned to any of the ortholog groups for the multi-gene phylogenetic analysis. In the transcriptome assemblies of *Dendroctonus frontalis* (TSA prefix GAFI01) and *Ixodes ricinus* (TSA prefix GADI01), we found multiple overlapping, yet clearly distinct, sequences of the same single-copy genes. As this indicates the presence of multiple parasite species in the sequenced sample, we also removed these assemblies from the phylogenetic analyses. In the following, we will focus on the 32 assemblies for which orthologous sequences were identified that putatively derived from a single parasite species. We also found overlapping sequences in some of the remaining assemblies. However, in these cases, the sequences were 100% identical in the overlapping regions but differed in length. We assume that poor sequence coverage of the parasite genes may have resulted in fragmented assemblies, though we cannot rule out haplotype variation or the presence of multiple, very closely related parasite species; neither of which should have an effect on the results of our phylogenetic analyses.

### The efficiency of curation of publicly available assemblies

The extracted sequence data may prove useful for researchers working on various aspects of parasite biology. The number of parasite-derived contigs in an assembly may depend on several factors, such as source tissue, parasitaemia, sequencing depth or pre- and post-assembly filtering methods to remove low-quality contigs or sequences of unknown origin. In this context, it should be noted that earlier versions of the genome assemblies from the western lowland gorilla, *Gorilla gorilla gorilla* (WGS prefix CABD02), and the platypus, *Ornithorhynchus anatinus* (WGS prefix AAPN01), which were employed in this study, contained large numbers of sequences that originated from apicomplexan parasites. Meanwhile, however, the majority of these contaminating sequences have been removed from the current assembly versions that are available in the public databases (WGS prefix CABD03 for the gorilla; contaminating contigs flagged as ‘dead’ in the AAPN01 record for the platypus).

Our analyses showed that the measures that were taken to remove off-target contigs were reasonably effective (98.0% of contaminants removed from the gorilla assembly and 91.5% from the platypus assembly). It is, of course, desirable that the final genome and transcriptome assemblies contain only high-quality contigs originating exclusively from the target species. However, we argue that the uncurated assemblies should also be made available to the research community because they constitute a valuable resource for data mining approaches and may allow us to gain insights into the pathogens infecting the target species.

### Phylogenetic classification of the contaminating parasites

**Fig. 3:**
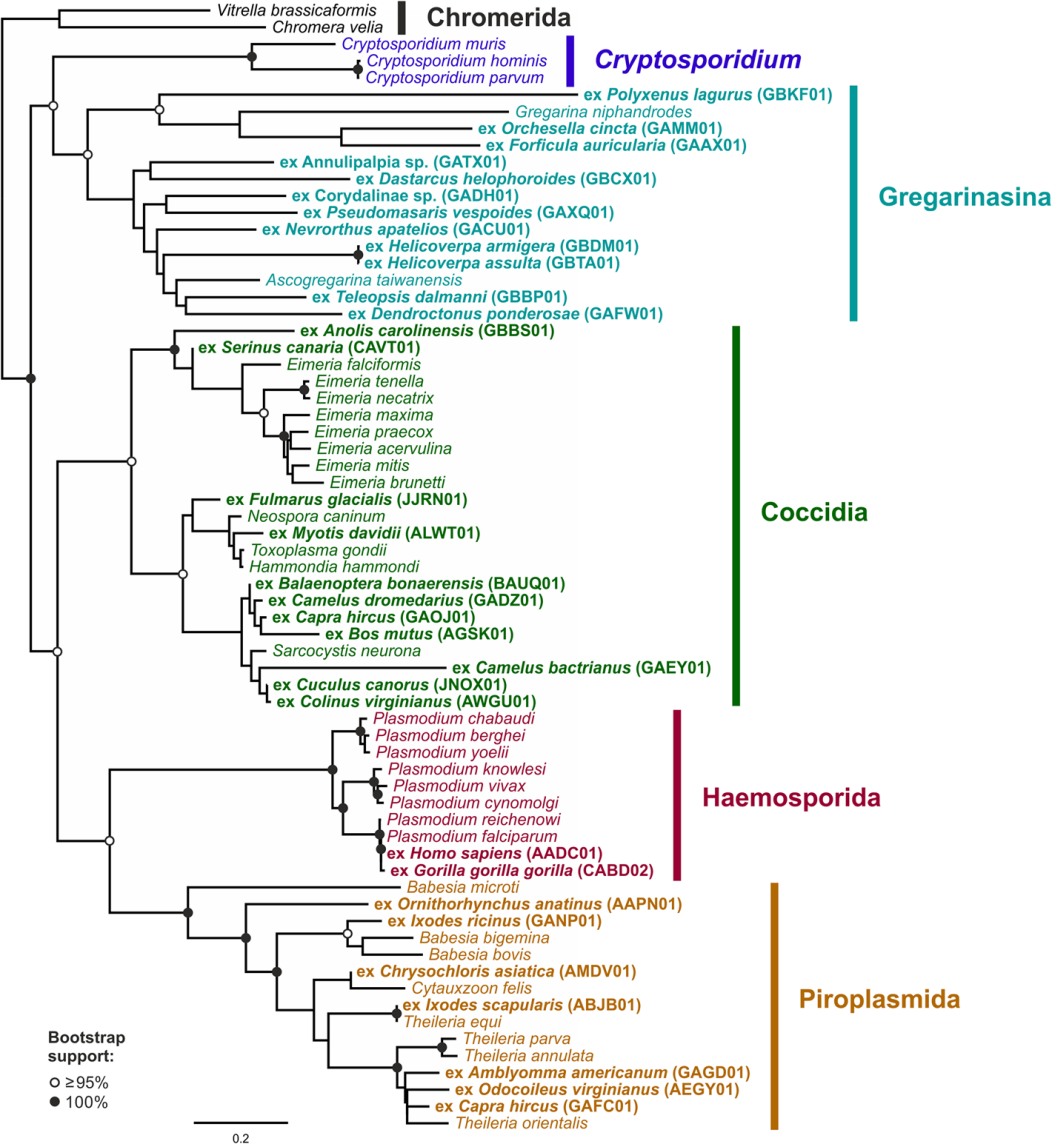
Maximum likelihood tree based on a RAxML analysis of dataset 1 (1,420 genes, 67 taxa). The tree was rooted with Chromerida

To understand the phylogenetic origin of the contaminating parasites, the extracted amino acid sequences were assigned to ortholog groups and used in a multi-gene phylogenetic analysis. The final dataset comprised 1,420 genes from 32 parasite contaminations and 35 previously sequenced apicomplexan and chromerid genomes (dataset 1). The phylogenetic analysis identified the contaminating parasites in the metazoan genome and transcriptome assemblies as members of the apicomplexan taxa Gregarinasina, Coccidia, Piroplasmida and Haemosporida (Fig. 3).

Contaminations by gregarine parasites were found in 12 assemblies, all of which were derived from arthropod transcriptomes. This observation is in line with gregarine life history, as these parasites are only found in invertebrate hosts [35]. Due to the lack of medical or veterinary importance of Gregarinasina, this taxon has essentially been neglected in genome sequencing efforts. Only a single gregarine draft genome is available from *Gregarina niphandroides* and a highly fragmented assembly from *Ascogregarina taiwanensis* that was estimated to contain 25% of the parasite’s genome. Yet, Gregarinasina constitute a key taxon for understanding the evolutionary history of Apicomplexa because of their basal position within the phylum. The extracted contaminating contigs significantly increase the amount of available sequence data from gregarine parasites and may prove to be a valuable resource for researchers studying the molecular evolution of these parasites.

In 11 assemblies from vertebrates, we identified contaminations by coccidian parasites, including the previously described contaminations in the genomes of *Myotis davidii* and *Colinus virginianus* [9]. In that study, the contaminations were identified by searching for a gene (apicortin) that is specific for apicomplexan parasites but absent from metazoan genomes. This method requires only few computational resources and is unlikely to produce false positives, as any significant hit is a clear indication of contamination. A similar methodology has recently been employed to identify sequences originating from insect pests in plant transcriptomes [10]. However, such an approach is bound to miss a large number of contaminations as it relies on a small, specific set of genes to be present in the (incomplete) assembly. Additionally, conserved genes which are suitable for deep-level phylogenetic analyses are rarely specific to a certain clade and often have homologs in extremely distantly related taxa. By targeting the whole parasite proteome, we are able to overcome these limitations for the identification and extraction of contaminating sequences.

In the assemblies of a human genome (WGS prefix AADC01) and the genome of the western lowland gorilla (WGS prefix CABD02), we found sequences that are ≥99.9% identical at the nucleotide level to sequences from the most virulent agent of human malaria, *Plasmodium falciparum*. The complete mitochondrial genome of the parasite is present in the superseded version of the gorilla genome assembly (EMBL/Genbank acc. nos. CABD02435943 and CABD02435942). The sequences are clearly more closely related to those from *P. falciparum* than to those from any known ape-infecting parasite (Additional file 1: Figure S1), including the *P. falciparum*-like parasites that have been reported from western lowland gorillas [36]. Additionally, exposure to parasites from wild gorillas seems implausible considering that the animal was born and raised in a North American zoo [37]. We, therefore, conclude that contamination with parasite DNA in the lab or at the sequencing center is the likely explanation in this case, though we cannot formally rule out an infection of the gorilla with *P. falciparum*. Taking into account that all other host-parasite associations that we found fit well with parasite biology (i.e. gregarines only in invertebrates, piroplasmids in tick vectors and vertebrate hosts), we consider infection of the sequenced organism as the most likely source of parasite contamination in the other assemblies.

Contaminations with piroplasmid parasites were found in the assemblies of tick vectors (*Amblyomma americanum*, *Ixodes ricinus*, *Ixodes scapularis*), as well as in putative vertebrate hosts (*Chrysochloris asiatica*, *Capra hircus*, *Odocoileus virginianus*, *Ornithorhynchus anatinus*). A recent study by Paparini et al. [38] has provided the first molecular data from *Theileria ornithorhynchi*, a piroplasmid parasite of the platypus. In a blastn search of piroplasmid 18S rRNA sequences against the platypus genome assembly [39], we identified a contig of piroplasmid origin encoding a fragment of the parasite’s 18S rRNA (EMBL/Genbank acc. nr. AAPN01188453). A phylogenetic analysis based on the dataset of Paparini et al. [38] indeed recovered this contig closely associated with the sequences from *T. ornithorhynchi* (Additional file 2: Figure S2). We also found a small number of sequences derived from a piroplasmid parasite in the genome assembly of the Cape golden mole (*Chrysochloris asiatica*; WGS prefix AMDV01). To the best of our knowledge, this is the first report of a piroplasmid infection in mammals belonging to the order Afrosoricida. The extracted sequences from the genome assembly of the blacklegged tick, *I. scapularis*, are identical to sequences from the equine parasite *Theileria equi*. While *I. scapularis* has not been described as a vector of this species, the sequenced ticks were fed on sheep [40], which may be natural hosts of *T. equi* [41]. However, a cautionary note is required: The presence of parasite DNA in the blood or tissue of a putative host indicates that the animal is naturally subjected to the parasite and that the parasite can develop in the host, but it does not prove that the parasite is able to complete its complex life cycle within the host and infect a new host.

### Deep phylogeny of Apicomplexa

The advent of molecular phylogenetics has challenged several longstanding views on the relationships among apicomplexan taxa, such as the monophyly of *Plasmodium* parasites [42, 43] or the inclusion of *Cryptosopidium* in Coccidia [44, 45]. The deep-level phylogenetic relationships of our tree are in good agreement with the current view on apicomplexan phylogeny. Like previous molecular studies [44, 46], we found a sister group relationship between *Cryptosporidium* and the gregarines at the base of Apicomplexa. Both parasite taxa appear to have lost their plastid genomes [47, 48] and also share numerous molecular similarities [46]. Piroplasmida and Haemosporida were united in a clade to the exclusion of Coccidia. Within Piroplasmida, *Babesia* was found to be paraphyletic – a finding that is congruent with the results of Schnittger et al. [49], who inferred six major monophyletic piroplasmid lineages based on all available 18S rRNA data. The authors concluded that a robust phylogeny based on multi-gene data might be required before re-interpretation of traditional characters could reconcile morphological and molecular data. A recent study on the phylogenetic relationships of *Theileria ornithorhynchi*, a parasite of the monotreme platypus, placed this species outside the clade of the theilerids and basal to all other piroplasms [38]. However, the results were inconclusive as this relationship was only recovered in the analysis of 18S rRNA data, while tree inference using the heat shock protein 70 (Hsp70) resulted in a markedly different phylogeny. Dataset 1 contains data from the platypus parasite for 540 orthologous genes. The resulting tree supported the tentative placement of *Theileria ornithorhynchi* based on 18S rRNA with maximum support. We found good support (92% bootstrap support) for a placement of the afrosoricid parasite extracted from *Chrysochloris asiatica* within the clade comprising all other *Theileria* parasites and *Cytauxzoon*. However, due to the low amount of data available for this species (only two genes present in dataset 1), its exact phylogenetic position remains unresolved (Fig. 3).

**Fig. 4:**
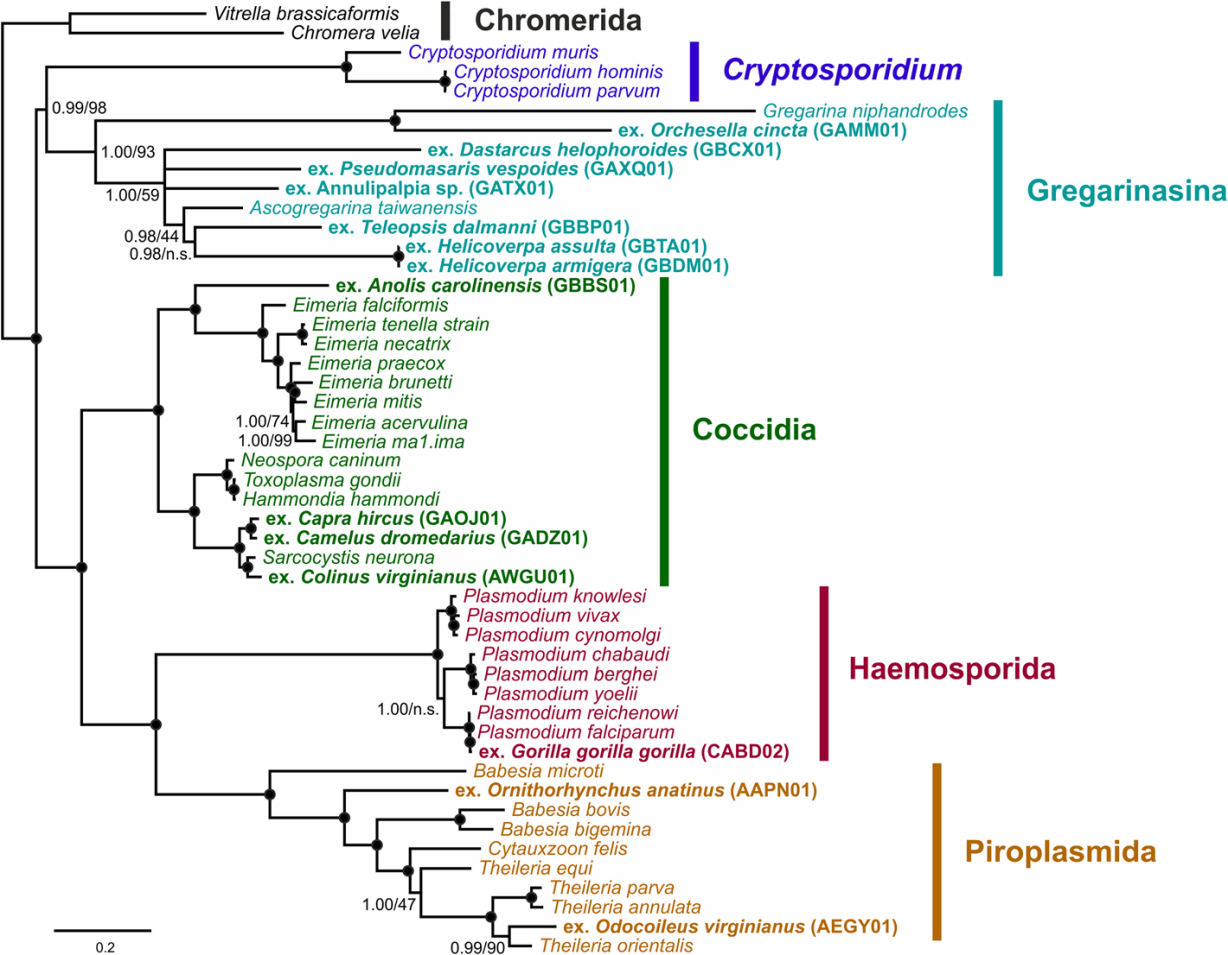
Majority-rule consensus tree based on a PhyloBayes analysis of dataset 2 (301 genes, 49 taxa). Bootstrap support values from a RAxML analysis were mapped onto the tree topology. Bayesian posterior probabilities < 1.00 and bootstrap support values < 100% are given at the nodes, respectively; n.s.: split was not supported in the ML analysis; splits that have 1.00 posterior probability and 100% bootstrap support are denoted by a dark circle. The tree was rooted with Chromerida

Phylogenetic analyses based on a reduced dataset that only contains the genes and taxa with the highest coverage (dataset 2) yielded a tree that is fully congruent with the results from the first analysis but with maximum support for nearly all splits (Fig. 4). This indicates that the reduced support for some deep-level splits in the first analysis is not due to conflict in the phylogenetic signal but rather due to the unstable positioning of some taxa with very low gene coverage.

## Conclusion

We were able to extract 20,907 parasite-derived contigs from 51 publicly available genome and transcriptome assemblies employing a new bioinformatic pipeline. Our results show that contaminations in sequencing data are not just a problem that needs to be eliminated but that they also constitute a valuable, cost-efficient source of information. Analysis of contaminations may enable the discovery and identification of novel parasite taxa and shed light on previously unknown host-parasite interactions. Our approach is not only valid for the identification of apicomplexan parasites but can also be used to study contaminations by other pathogens, such as bacteria or viruses. Most genomic and transcriptomic studies only make the raw sequencing data and the final curated and annotated assemblies available to the public. While these datasets are obviously most relevant to and useful for the subject of study, we argue that uncurated assemblies may contain valuable information from unexpected sources and should, therefore, routinely be made available.

## Additional files

Additional file 1: Figure S1. Majority-rule consensus tree based on a PhyloBayes analysis of complete mitochondrial genomes from ape-infecting *Plasmodium* parasites. The alignment is based on the mitochondrial dataset from Liu et al. (2010) and only contains sequences from Clades C1 (from Chimpanzees) and G1 (from Gorillas; also contains human *P. falciparum*). Two contigs from the Gorilla genome assembly, which contain parasite-derived mitochondrial fragments, were added to the alignment. Bayesian posterior probabilities are given at the nodes. The tree was rooted with the C1 clade of Chimpanzee-infecting *Plasmodium* parasites. All EMBL/Genbank acc. nos. are given in parentheses. (PDF 236 kb)
Additional file 2: Figure S2. Majority-rule consensus tree based on a PhyloBayes analysis of 18 s rRNA sequences from Piroplasmida. The alignment is based on the 18 s dataset from Paparini et al. (2015). A single contig from the platypus genome assembly, which contains a parasite-derived 18 s rRNA fragment, was added to the alignment. Bayesian posterior probabilities are given at the nodes. The tree was rooted with *Cardiosporidium cionae*. All EMBL/Genbank acc. nos. are given in parentheses. (PDF 157 kb)

## Abbreviations

Aa: Amino acid
EuPathDB: Eukaryotic Pathogen Database
Gb: Giga base pairs
Hsp70: Heat shock protein 70
Mb: Mega base pairs
ML: Maximum likelihood
NGS: Next-generation sequencing
TSA: Transcriptome Shotgun Assembly
WGS: Whole Genome Shotgun
