# Supplementary material for: Parasite infection of public databases: a data mining approach to identify apicomplexan contaminations in animal genome and transcriptome assemblies
Source: BMC Genomics. 2017 Jan 19;18:100. doi: 10.1186/s12864-017-3504-1 (PMC5244568; doi:10.1186/s12864-017-3504-1)
Supplement: Additional file 1: Figure S1. — Majority-rule consensus tree based on a PhyloBayes analysis of complete mitochondrial genomes from ape-infecting Plasmodium parasites. The alignment is based on the mitochondrial dataset from Liu et al. (2010) and only contains sequences from Clades C1 (from Chimpanzees) and G1 (from Gorillas; also contains human P. falciparum). Two contigs from the Gorilla genome assembly, which contain parasite-derived mitochondrial fragments, were added to the alignment. Bayesian posterior probabilities are given at the nodes. The tree was rooted with the C1 clade of Chimpanzee-infecting Plasmodium parasites. All EMBL/Genbank acc. nos. are given in parentheses. (PDF 236 kb) [file 12864_2017_3504_MOESM1_ESM.pdf]

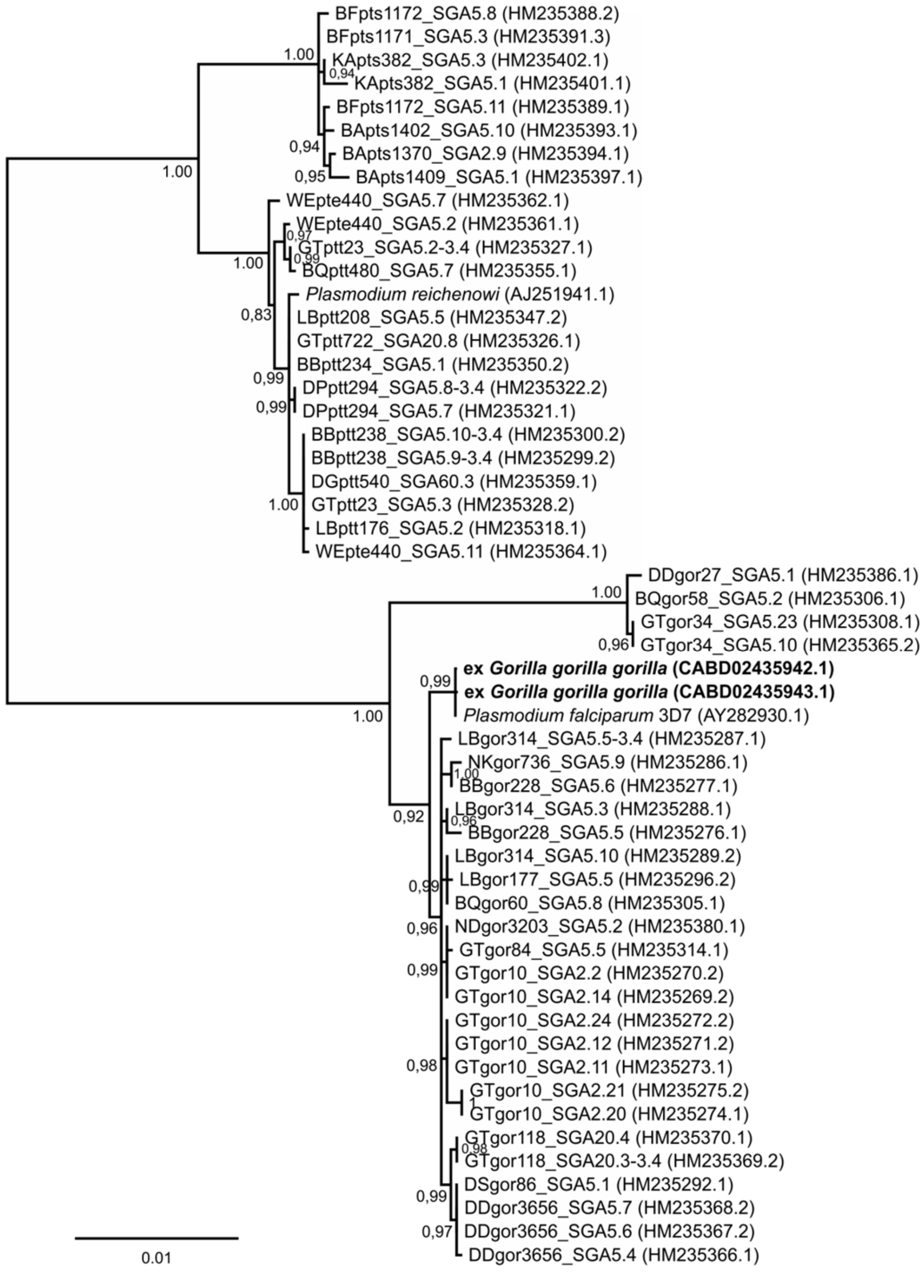

Additional file 1: Figure S1. Majority-rule consensus tree based on a PhyloBayes analysis of complete mitochondrial genomes from ape-infecting *Plasmodium* parasites. The alignment is based on the mitochondrial dataset from Liu et al. (2010) and only contains sequences from Clades C1 (from Chimpanzees) and G1 (from Gorillas; also contains human *P. falciparum*). Two contigs from the Gorilla genome assembly, which contain parasite-derived mitochondrial fragments, were added to the alignment. Bayesian posterior probabilities are given at the nodes. The tree was rooted with the C1 clade of Chimpanzee-infecting *Plasmodium* parasites. All EMBL/Genbank acc. nos. are given in parentheses.
