# Supplementary material for: Parasite infection of public databases: a data mining approach to identify apicomplexan contaminations in animal genome and transcriptome assemblies
Source: BMC Genomics. 2017 Jan 19;18:100. doi: 10.1186/s12864-017-3504-1 (PMC5244568; doi:10.1186/s12864-017-3504-1)
Supplement: Additional file 2: Figure S2. — Majority-rule consensus tree based on a PhyloBayes analysis of 18 s rRNA sequences from Piroplasmida. The alignment is based on the 18 s dataset from Paparini et al. (2015). A single contig from the platypus genome assembly, which contains a parasite-derived 18 s rRNA fragment, was added to the alignment. Bayesian posterior probabilities are given at the nodes. The tree was rooted with Cardiosporidium cionae. All EMBL/Genbank acc. nos. are given in parentheses. (PDF 157 kb) [file 12864_2017_3504_MOESM2_ESM.pdf]

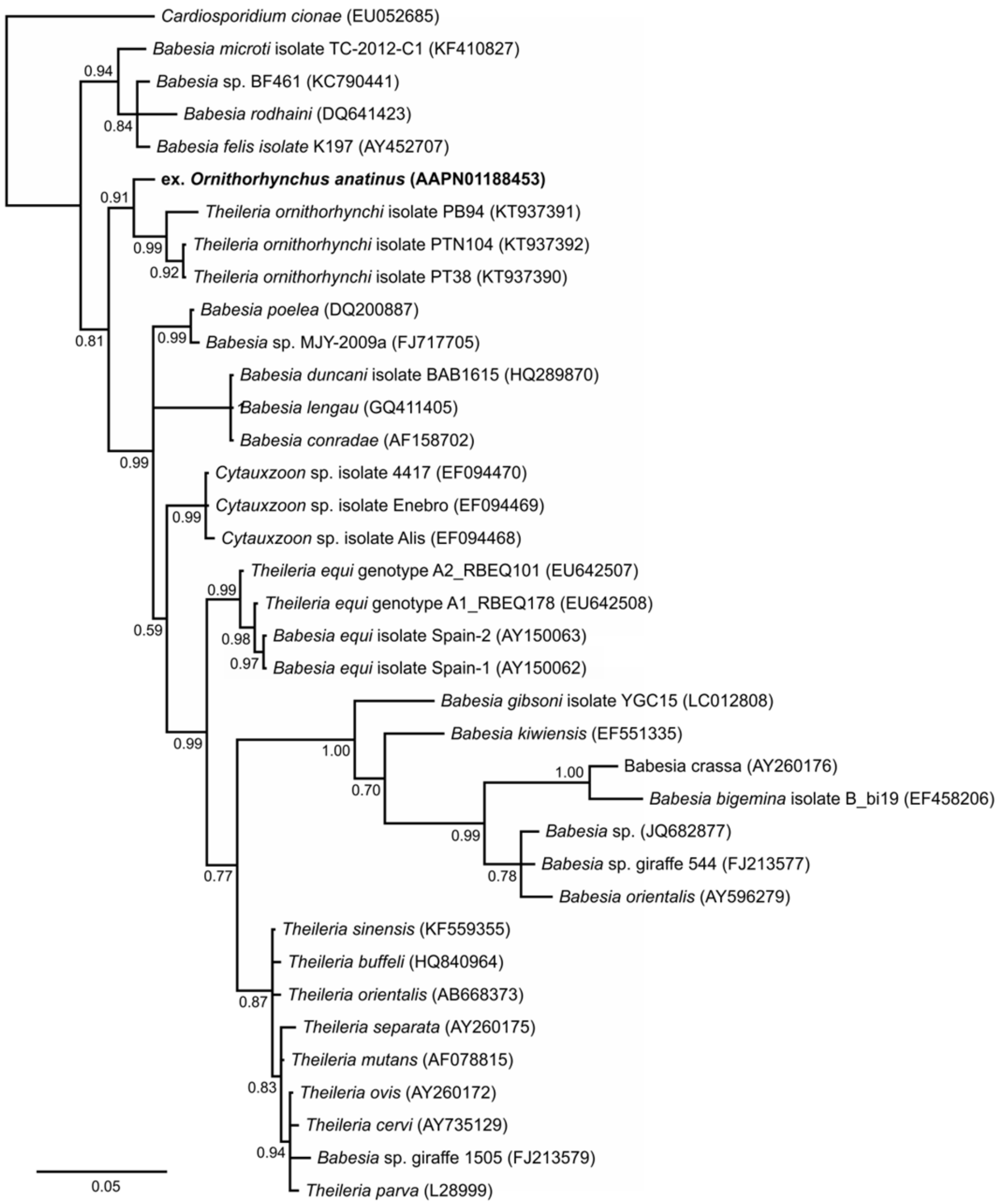

Additional file 2: Figure S2. Majority-rule consensus tree based on a PhyloBayes analysis of 18s rRNA sequences from Piroplasmida. The alignment is based on the 18s dataset from Paparini et al. (2015). A single contig from the platypus genome assembly, which contains a parasite-derived 18s rRNA fragment, was added to the alignment. Bayesian posterior probabilities are given at the nodes. The tree was rooted with *Cardiosporidium cionae*. All EMBL/Genbank acc. nos. are given in parentheses.
